# Blueberry Consumption Affects Serum Uric Acid Concentrations in Older Adults in a Sex-Specific Manner

**DOI:** 10.3390/antiox5040043

**Published:** 2016-11-29

**Authors:** Carol L. Cheatham, Itzel Vazquez-Vidal, Amanda Medlin, V. Saroja Voruganti

**Affiliations:** 1 Department of Psychology & Neuroscience, University of North Carolina at Chapel Hill, Chapel Hill, NC 27599, USA; 2 Nutrition Research Institute, University of North Carolina at Chapel Hill, 500 Laureate Way, Rm 1101, Kannapolis, NC 28081, USA; itzel_vazquez@unc.edu (I.V.-V.); saroja@unc.edu (V.S.V.); 3 University of North Carolina at Charlotte, Charlotte, NC 28223, USA; amedli13@uncc.edu; 4 Department of Nutrition, University of North Carolina at Chapel Hill, Chapel Hill, NC 27599, USA

**Keywords:** hyperuricemia, blueberries, cardiovascular disease, antioxidant

## Abstract

Blueberries are rich in antioxidants and may protect against disease. Uric acid accounts for about 50% of the antioxidant properties in humans. Elevated levels of serum uric acid (SUA) or hyperuricemia is a risk factor for cardiovascular disease (CVD). The aim was to determine the effect of blueberries on SUA in older adults. Participants (*n* = 133, 65–80 years) experiencing mild cognitive impairment (MCI) were randomized in a double-blind 6-month clinical trial to either blueberry or placebo. A reference group with no MCI received no treatment. The mean (SD) SUA at baseline were 5.45 (0.9), 6.4 (1.3) and 5.8 (1.4) mg/dL in reference, placebo, and treatment groups, respectively. Baseline SUA was different in men and women (6.25 (1.1) vs. 5.35 (1.1), *p* = 0.001). During the first three months, SUA decreased in the blueberry group and was significantly different from the placebo group in both men and women (*p* < 0.0003). Sex-specific differences became apparent after 3 months, when only men showed an increase in SUA in the blueberry group and not in the placebo (*p* = 0.0006) between 3 and 6 months. At 6 months SUA had rebounded in both men and women and returned to baseline levels. Baseline SUA was correlated with CVD risk factors, waist circumference and triglycerides (*p* < 0.05), but differed by sex. Overall, 6 m SUA changes were negatively associated with triglycerides in men, but not in women. Group-wise association between 6 m SUA changes and CVD risk factors showed associations with diastolic blood pressure, triglycerides and high-density lipoprotein (HDL) cholesterol in women of the Blueberry group but not in men or any sex in the placebo group. In summary, blueberries may affect SUA and its relationship with CVD risk in a sex-specific manner.

## 1. Introduction

Uric acid accounts for more than half of the antioxidative properties in humans [1,2,3,4]. However, elevated serum uric acid concentrations (SUA) or hyperuricemia is considered a risk factor for the development of oxidative stress and/or inflammation-related diseases such as gout, hypertension, cardiovascular disease (CVD) and chronic kidney disease (CKD) [1]. Uric acid has a paradoxical role in human metabolism: on one hand, it acts as an antioxidant in hydrophilic environment, whereas on the other hand, it can propagate a chain reaction and cause oxidative damage to cells in reaction with other radicals [1]. However, the role of SUA in CVD remains controversial with conflicting information being put forward regarding the link between SUA and CVD [5,6,7].

Plant polyphenols have been shown to be protective against diseases such as certain cancers, CVD, diabetes, and aging [8,9,10]. Blueberries contain anthocyanins, which are recognized to possess antioxidant and anti-inflammatory properties. Human and animal studies have shown beneficial effects of blueberry consumption on lifestyle-related chronic diseases [11]. Studies have been conducted to understand the effect of polyphenol and anthocyanins containing foods on circulating uric acid concentrations. On the one hand, red wine has been shown to acutely increase plasma urate concentrations [12], whereas, on the other hand, dealcoholized red wine has been shown to decrease plasma uric acid concentrations [13]. Similarly, cherry consumption has been shown to reduce SUA concentrations whereas acaiberry had no effects on them [14,15]. Although, to the best of our knowledge, blueberries have not been tested for their effects on SUA and no scientific evidence links them so far, they are thought to be functional foods for curing gout, an inflammatory arthritic disease caused by hyperuricemia, mainly for their anthocyanin content or antioxidant property.

The role of berry fruits in improving cardiovascular health is gaining importance [8]. Studies have shown beneficial effects of flavonoids and anthocyanins on endothelial function, type 2 diabetes, and hypertension. A study in 93,600 young to middle-aged women from the Nurses Health Study (NHS) II showed that high intake of anthocyanins can help reduce risk for myocardial infarction [16]. Another study from the same group also showed that anthocyanins and specific flavones can prevent hypertension [17] and decrease the risk for type 2 diabetes [18]. In a randomized, double-blind placebo-controlled clinical trial, blueberry consumption was associated with improvement in endothelial function, but not in blood pressure, in 44 adult men [19]. In contrast, in another double-blind placebo-controlled clinical trial, blueberries were found to decrease blood pressure and arterial stiffness in 8 weeks in postmenopausal women [20]. Additionally, a dose and time-dependent improvement was observed in vascular function following blueberry consumption in men participating in a double-blind, crossover intervention study [21]. However, very few studies have investigated the effect of blueberry consumption on SUA concentration or its relationship with CVD risk factors. A literature review revealed only one study which was conducted in 15 healthy adults that did not find any significant differences in SUA in individuals with high, low, or no dose blueberries [22].

Thus, the aim of this study was to determine the impact of blueberries on SUA concentrations and their relation to CVD risk factors in older adults. Our central hypothesis is that the blueberries, when added to the diet, will enhance the body’s antioxidant status, lower SUA concentrations, and improve related CVD risk factors.

## 2. Research Design and Methods

### 2.1. Study Population

In the context of a larger study that was designed to assess the effects of blueberries on cognitive abilities, adults aged 65 to 80 years, who were beginning to experience mild cognitive decline, but were generally healthy, were enrolled in a 6-month double-blind randomized clinical trial across a 3-year rolling enrollment. Inclusion criteria included those who consumed fewer than five daily servings of fruits and vegetables; were not diagnosed with dementia or Alzheimer’s disease, central nervous system disorders, psychiatric disorders, gastrointestinal issues, digestive issues, or diabetes; had a body mass index (BMI) less than 34.9 lbs./in^2^; were not taking certain medicines (e.g., medications with known cognitive side-effects); and were right-handed. The study was approved by the Institutional Review Board for the University of North Carolina at Chapel Hill, and all participants gave written and verbal informed consent (11-2075).

**Figure 1:**
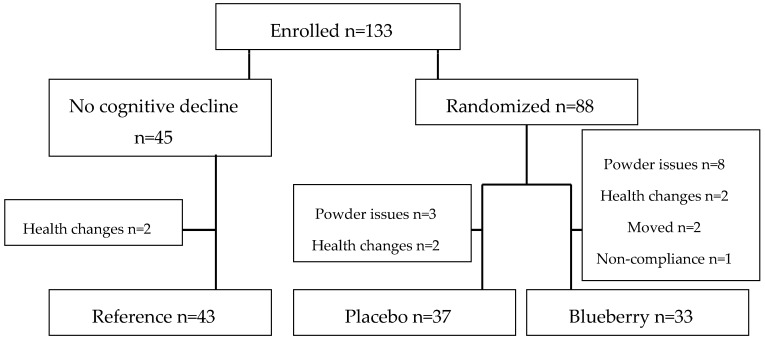
A Consort diagram of the participant flow of the parent clinical trial.

Intervention participants were randomly assigned to either the blueberry or placebo group (Figure 1). Participants were age-matched in half-decade categories (65–69, 70–74, and 75–79 years old). Single-year-crop wild blueberries donated by the Wild Blueberry Association of North America (Old Town, ME, USA) were freeze-dried and pulverized into a powder at Futureceuticals, Inc. (Momence, IL, USA) and packaged in 17.5 g packets at WePackItAll (Irwindale, CA, USA). The placebo powder was created by Futureceuticals from a recipe developed by the High Bush Blueberry Council and was also packaged at WePackItAll. The packaging was blank with the exception of one of four letters (C, D, E, or F), which constituted the blind. Powders were analyzed for nutrient content and antioxidant activity by Medallion Labs (Minneapolis, MN, USA) at least semi-annually during the study to insure the powders maintained consistency over time (see Table 1 for nutrient composition).

Participants were instructed to consume two packets of powder (35 g)—which for the blueberries was the equivalent of two cups of fresh blueberries—daily for 180 days. Participants were further instructed that the powders were not to be heated, cooked, or added to already hot foods; it was also recommended that they not mix the powder with dairy products [23,24]. Participants recorded powder consumption times and details in a provided food diary. Any packets not consumed were returned to researchers; compliance was calculated from returned packets.

Participants were interviewed about the foods listed in their diary using a 3-pass diet recall system. Data were collected in a standardized format with the aid of food models for accurate portion determination utilizing the Nutrition Data System for Research (NDSR) software (Minneapolis, MN, USA). The NDSR software calculates average daily caloric and nutrient intake.

### 2.2. Uric Acid

Twelve-hour fasted blood was obtained from participants at baseline, 3 months, and 6 months. Blood was collected by a trained phlebotomist through blood draw using ethylenediaminetetraacetic acid (EDTA) coated tubes and serum tubes. Blood was centrifuged for 15 min at 1500*g* at 4 °C; layers were aliquoted into separate tubes and immediately stored at −80 °C. The blood was processed by Carolinas Medical Center LabCorp (Charlotte, NC, USA); the blood was assayed for creatinine, glucose, cholesterol, triglycerides, high-density lipoprotein (HDL) cholesterol and low-density lipoprotein (LDL) cholesterol.

### 2.3. Physical Examination

Anthropometrics and blood pressure were collected from participants at each session by trained research assistants. After participants sat quietly for five min with both feet on the floor, blood pressure was measured using an Omron HEM-907XL digital blood pressure monitor. Waist circumference was measured 1 inch above the navel using a soft tape measure. Participants’ weights and heights were measured without shoes and after emptying pockets and removing excess clothing. The Cardinal Detecto Pro Doc Series digital physician scale was used to measure weight. Height was measured to the nearest quarter inch using a Charder portable stadiometer situated against the wall.

### 2.4. Statistical Analysis

All statistical analyses were computed using STATA, version 14.0 (StataCorp LP, College Station, TX, USA). A test of normality was performed and the variables showing skewed distribution were log transformed to meet the normal distribution. *t*-tests and ANOVA were used to analyze group and sex differences. Pearson correlation tests and multiple linear regression analyses were used to assess the relationship between SUA concentrations and CVD risk factors. A value of <0.05 (two-tailed) was considered to be statistically significant after adjustment for multiple tests.

## 3. Results

A total of 58 men and 75 women aged 65 to 80 years participated in the larger study. Of the 133 enrolled participants, 107 provided blood samples and were included in the analysis: 47 men and 60 women. Their descriptive characteristics are depicted in Table 2. The 6-month period treatment showed no differences in body weight and waist circumferences in any of the three groups. No other metabolic risk factors showed any significant changes across the 6-month period.

### 3.1. Compliance

In both treatment groups, although not significant, women seem to be more compliant with powder consumption than men. In the placebo group, compliance was 93% and 81% in men at three and six months, respectively, whereas it was 97% in women at both the three- and six-month timepoints. In the blueberry group, however, compliance was lower at 78% and 70% in men at three and six months, respectively and 81% and 62% in women at three and six months, respectively.

### 3.2. Changes in SUA Following Supplementation with Blueberries or Placebo

**Figure 2:**
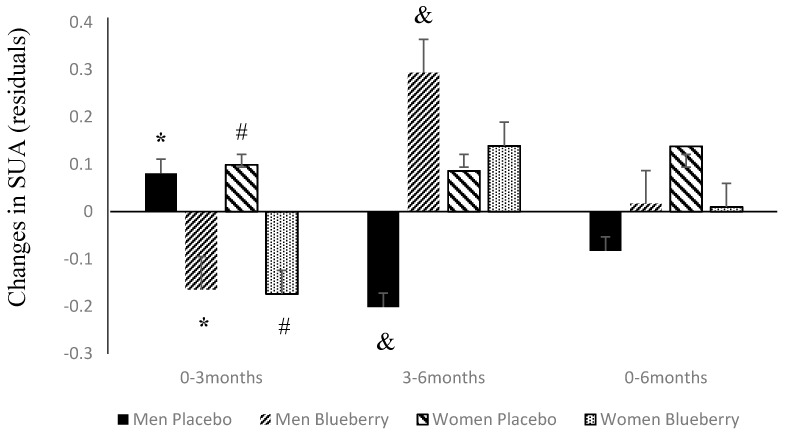
SUA (serum uric acid) changes over 6 months according to sex and treatment group. Residual values are shown as mean (SD); * *p* = 0.0002, # *p* = 0.00001, & *p* = 0.0006.

At baseline mean (SD) SUA, levels were 5.91 (1.3) mg/dL. When categorized by sex and treatment groups, SUA concentrations were higher in men than women (6.25 (1.1) vs. 5.35 (1.1) mg/dL, *p* = 0.001) but not significantly different between placebo and treatment groups (6.1 (1.2) vs. 5.8 (1.4), *p* = 0.43) at baseline. Changes in SUA between baseline and 3 months (0–3 months), 3 to 6 months (3–6 months) and baseline to 6 months (0–6 months) were adjusted for age, waist circumference, total calorie intake and blueberry intake compliance and residuals were used for further analysis. Figure 2 depicts the differences in these changes between the two sexes and groups. SUA during 0–3 months decreased significantly in the blueberry group as compared to the placebo group in both men (*p* = 0.0002) and women (*p* = 0.00001). However, during 3–6 months, in men, SUA increased significantly in the blueberry group as compared to the placebo group (*p* = 0.0006) but not in women (*p* = 0.87). By the end of 6 months, SUA had decreased to its baseline levels and was not significantly different between groups in both men (*p* = 0.23) and women (*p* = 0.54).

### 3.3. Changes in SUA and Its Relation to Changes in Other CVD Risk Factors

Regardless of sex differences, baseline SUA concentration was significantly correlated with waist circumference (*r* = 0.36, *p* < 0.001), triglycerides (*r* = 0.31, *p* < 0.01), glucose (*r* = 0.27, *p* < 0.01), and HDL cholesterol (*r* = −0.43, *p* < 0.001). Sex-specific significant correlations of SUA concentrations were observed with triglycerides (*r* = 0.30, *p* < 0.05) in men and with waist circumference (*r* = 0.50, *p* < 0.0001), triglycerides (*r* = 0.42, *p* < 0.001), glucose (*r* = 0.45, *p* = < 0.001), and HDL cholesterol (*r* = −0.42, *p* = 0.001) in women. Changes in SUA concentrations (baseline to 6 months) were positively correlated with glucose (*r* = 0.32, *p* = 0.03) and negatively with systolic (*r* = −0.36, *p* = 0.009) and diastolic blood pressure (*r* = −0.36, *p* = 0.009) in women (data not shown in tables). Multiple regression, adjusted for blueberry compliance, showed the baseline SUA concentrations were significantly correlated with CVD risk factors, waist circumference and serum triglycerides (*p* < 0.05). However, the association of changes in SUA concentrations differed by sex and treatment. Changes in SUA concentrations were negatively associated with blood glucose and triglycerides in men whereas no significant associations were observed in women (Table 3).

When categorized by treatment groups (blueberry vs. placebo), changes in SUA concentrations were not associated with any CVD risk factors in the placebo group. Conversely, in the blueberry group, changes in SUA concentrations were significantly associated with diastolic blood pressure, and serum levels of triglycerides and HDL cholesterol and showed suggestive evidence of association with changes in total cholesterol in women but not in men (Table 4). At baseline, SUA, total cholesterol and changes in SUA and total cholesterol were similar in both sexes in the placebo group. However, in the blueberry group, beta coefficient was in opposite direction for association between changes in SUA and diastolic blood pressure, triglycerides, HDL and total cholesterol between men and women. Thus, there seems to be an effect modification by sex in the relationship of SUA with these CVD risk factors.

## 4. Discussion

In the present study, we investigated the effects of blueberry consumption on SUA levels and its relationship with CVD risk factors in older adults. Uric acid, a major antioxidant in humans, can also act as pro-oxidant and increase the risk for major chronic diseases such as hypertension, type 2 diabetes, CVD, and CKD thus has contradictory effects on metabolism. Elevated SUA concentrations have been associated with increased risk for all-cause and CVD mortality [25]. A number of studies have investigated the role of uric acid in the development of CVD, however, very limited studies have been conducted in older adults. Moreover, no studies have investigated the effect of nutritional intervention (blueberry) on SUA concentrations in older individuals. Considering that both have anti-oxidative properties, it is important that we understand the effect of blueberry supplementation on SUA concentrations.

Our results show that, at baseline, men had significantly higher levels of SUA than women. Significant differences have been observed in the levels of SUA concentrations in men and women. Several studies have reported higher levels of SUA in men as compared to women [26,27,28,29,30,31,32,33], which is similar to our results. Similarly, sex-specific differences have also been reported with respect to association of SUA with CVD risk factors. Chou et al. [34] conducted a stratified analysis and found association of SUA with insulin resistance and plasma glucose levels in women but not in men. Previous studies from our group and others have shown a positive association between SUA and waist circumference [27,28,32,34]. In the current study, we found a strong correlation between SUA and waist circumference at baseline, mainly in women.

Many studies have observed the association between CVD risk factors and SUA concentrations to be stronger in women than men [32,33,35]. We also observed strong correlations of baseline SUA with serum triglycerides. This, again, is a replication of observations reported by several studies [32,35,36,37]. A retrospective analysis of the database from the Laboratory Information System—a database of data from a cohort of outpatient adults referred by general practitioners for routine medical check-ups for three years from 2005–2008—showed that triglycerides were significantly associated with circulating uric acid in women but not in men [35]. Similarly, a study conducted in elderly adults reported that high SUA levels predicted metabolic syndrome in older women but not men [31]. In our study, we observed a similar pattern of associations. Changes in SUA between baseline and the 6-month time-period were significantly associated with CVD risk factors (glucose and triglycerides) in men but not women. Changes in SUA regardless of treatment group also showed sex-specific differences in associations between SUA and CVD risk factors. Thus, our study replicates and confirms that the association of baseline SUA and changes in SUA are significantly associated with some CVD risk factors in a sex-specific manner.

As a next step, we investigated whether blueberry consumption affected SUA concentrations. We found that SUA concentrations initially decreased regardless of sex differences. However, after the 3-month time point, sex-specific differences became apparent. Significant differences between placebo and blueberry groups between 3 and 6 months were found only in men and not in women. Likewise, changes in SUA concentration were significantly associated with diastolic blood pressure, and serum levels of triglycerides and HDL cholesterol only in women but not men in the blueberry group. No significant associations or sex-specific differences were observed in the placebo group. However, the sex-specific differences in changes in SUA between and within groups are noteworthy. To the best of our knowledge, this is the first study to focus on the effects of blueberry on SUA concentrations over a 6-month long period. There is only one other study [22] in which scientists investigated the effects of high and low dose of blueberries on measures of antioxidant status and included SUA as one of the measures. However, this study measured only the acute effects (fasting, 1, 2, and 3 h after sample consumption) and did not find any significant differences in SUA concentrations. We, on the other hand, investigated the long-term effects of blueberry consumption. We also found association of blueberry compliance with SUA concentrations where increasing compliance was associated with deceasing SUA in women.

Our study has some limitations. Most importantly, our sample size may be too small to detect a significant effect: the study was not powered for a sex split. Despite the small size, we were able to find a significant association between changes in SUA, consumption of blueberries, and CVD risk factors. Now that the evidence for sex differences is clear, future studies should include sufficient numbers of participants to analyze by sex. In addition, eleven participants dropped out because of issues with the study powders. Thus, the results are only generalizable to those who can tolerate a diet with added berries and sugars. Finally, we didn’t have measures of individual markers of anti-oxidant status, and as a result, we could not evaluate the direct effect of blueberry consumption on anti-oxidant status.

## 5. Conclusions

In conclusion, blueberry consumption seems to affect SUA concentrations and its relationship with CVD risk factors in a sex-specific manner. Importantly, lowering of SUA with consumption of blueberry powder in women, and not men, warrants further studies to confirm and validate these results in a larger sample.

## Figures and Tables

**Table 1:** Average nutrition provided by powders per day if participants were 100% compliant.

Composition	Blueberry	Placebo
Calories	139	137
Saturated Fat (g)	0.084	0.000
Monounsaturated Fatty Acids (g)	0.138	0.000
Polyunsaturated Fatty Acids (g)	0.403	0.004
Trans Fatty Acids (g)	0.003	0.000
Cholesterol (g)	0.37	0.35
Sodium (mg)	2.60	7.46
Carbohydrates (g)	32.08	34.26
Fiber (g)	7.05	0.02
Fructose (g)	11.47	4.56
Glucose (g)	10.65	0.30
Sucrose (g)	0.05	0.04
Maltose (g)	0.07	0.54
Lactose (g)	0.04	0.04
Protein (g)	1.08	0.27
Calcium (g)	37.25	3.54
Iron (g)	0.54	0.05
Vitamin C (g)	9.34	0.25

**Table 2:** Descriptive statistics at baseline, 3 months, and 6 months.

Trait	Baseline	Mean (SD) 3 Months	6 Months
Age	72.68 ± 4.3		
Body weight (lb)	170.17 ± 31.7	171.43 ± 31.2	171.52 ± 31.5
Waist circumference (in)	39.60 ± 4.0	39.86 ± 4.2	39.61 ± 4.0
Systolic blood pressure (mmHg)	131.76 ± 17.3	129.47 ± 16.3	130.59 ± 16.9
Diastolic blood pressure (mmHg)	74.42 ± 9.1	73.68 ± 9.2	73.44 ± 10.9
Glucose (mg/dL)	100.22 ± 11.5	98.97 ± 13.3	99.88 ± 12.6
Triglycerides (mg/dL)	137.29 ± 97.7	131.28 ± 69.2	127.82 ± 64.4
Total cholesterol (mg/dL)	177.98 ± 34.4	179.48 ± 33.0	177.70 ± 36.7
LDL cholesterol (mg/dL)	98.50 ± 28.3	99.00 ± 27.0	97.10 ± 30.6
HDL cholesterol (mg/dL)	53.64 ± 16.0	54.73 ± 16.5	54.68 ± 16.5
SUA (mg/dL)	5.75 ± 1.3	5.21 ± 1.4	5.66 ± 1.3

LDL-low-density lipoprotein: HDL-high-density lipoprotein: SD-standard deviation; SUA-serum uric acid.

**Table 3:** Multiple regression analysis of baseline SUA concentrations and changes in SUA concentrations with cardiovascular disease risk factors.

Trait **	CVD Risk Factor	β (SE)	*t*	*p* Value *
Baseline—All	Waist circumference	0.073 (0.03)	2.67	0.009
	Glucose	0.009 (0.009)	0.96	0.34
	Systolic blood pressure	−0.003 (0.008)	−0.42	0.67
	Diastolic blood pressure	−0.0004 (0.01)	−0.03	0.98
	Triglycerides	0.003 (0.001)	2.31	0.023
	Total cholesterol	−0.003 (0.01)	0.44	0.66
	LDL cholesterol	0.005 (0.01)	−0.30	0.77
	HDL cholesterol	−0.010 (0.10)	0.39	0.24
Men	Waist circumference	0.0102 (0.06)	0.18	0.86
	Glucose	−0.002 (0.02)	−0.15	0.88
	Systolic blood pressure	−0.16 (0.01)	−1.27	0.21
	Diastolic blood pressure	0.24 (0.02)	1.08	0.29
	Triglycerides	0.002 (0.002)	1.51	0.14
	Total cholesterol	−0.002 (0.02)	−0.13	0.90
	LDL cholesterol	−0.002 (0.01)	0.12	0.91
	HDL cholesterol	−0.002 (0.02)	−0.15	0.88
Women	Waist circumference	0.085 (0.03)	2.72	0.009
	Glucose	0.018 (0.01)	1.33	0.19
	Systolic blood pressure	0.003 (0.01)	0.25	0.80
	Diastolic blood pressure	−0.173 (0.02)	−0.95	0.35
	Triglycerides	0.002 (0.01)	−0.78	0.44
	Total cholesterol	−0.004 (0.01)	−0.31	0.76
	LDL cholesterol	−0.008 (0.02)	0.51	0.61
	HDL cholesterol	−0.09 (0.1)	1.33	0.19
Changes over 6-month period **—All	Waist circumference	0.004 (0.1)	0.08	0.94
	Glucose	0.014 (0.01)	0.96	0.34
	Systolic blood pressure	−0.009 (0.01)	−0.84	0.41
	Diastolic blood pressure	−0.0003 (0.01)	−0.02	0.98
	Triglycerides	−0.0003 (0.002)	−0.17	0.87
	Total cholesterol	−0.008 (0.02)	−0.50	0.62
	LDL cholesterol	0.021 (0.02)	1.1	0.28
	HDL cholesterol	0.036 (0.02)	1.58	0.12
Men	Waist circumference	0.083 (0.05)	1.6	0.14
	Glucose	−0.045 (0.02)	−2.19	0.05
	Systolic blood pressure	−0.022 (0.02)	−1.33	0.21
	Diastolic blood pressure	0.022 (0.02)	1.23	0.24
	Triglycerides	−0.007 (0.002)	−3.21	0.008
	Total cholesterol	−0.0001 (0.02)	−0.01	0.99
	LDL cholesterol	0.009 (0.03)	0.34	0.74
	HDL cholesterol	0.070 (0.04)	1.81	0.098
Women	Waist circumference	−0.062 (0.07)	−0.87	0.40
	Glucose	0.033 (0.02)	2.02	0.06
	Systolic blood pressure	−0.005 (0.1)	−0.48	0.64
	Diastolic blood pressure	−0.033 (0.03)	−1.32	0.21
	Triglycerides	0.002 (0.005)	0.33	0.74
	Total cholesterol	−0.025 (0.02)	−1.32	0.20
	LDL cholesterol	0.042 (0.02)	−1.78	0.08
	HDL cholesterol	0.025 (0.02)	0.63	0.54

* all *p* values ≤ 0.06 are bolded; ** adjusted for age, compliance and total calorie intake. CVD: cardiovascular disease. SE: Standard error of mean.

**Table 4:** Multiple regression analysis of changes in SUA concentrations with cardiovascular disease risk factors in the treatment groups.

Trait **	CVD Risk Factor	β (SE)	*t*	*p* Value *
Placebo group—Men	Waist circumference	−0.036 (0.06)	−0.63	0.59
	Glucose	0.029 (0.14)	0.21	0.86
	Systolic blood pressure	−0.00009 (0.01)	0.02	0.99
	Diastolic blood pressure	0.006 (0.02)	0.29	0.80
	Triglycerides	0.0004 (0.001)	0.36	0.75
	Total cholesterol	−0.003 (0.008)	−0.40	0.73
	LDL cholesterol	−0.004 (0.007)	−0.59	0.57
	HDL cholesterol	−0.047 (0.05)	−0.86	0.48
Women	Waist circumference	−0.107 (0.08)	−1.37	0.21
	Glucose	0.0034 (0.01)	0.19	0.85
	Systolic blood pressure	−0.0105 (0.01)	−0.74	0.48
	Diastolic blood pressure	0.0031 (0.02)	0.14	0.90
	Triglycerides	0.0047 (0.007)	0.72	0.50
	Total cholesterol	−0.0163 (0.02)	−0.96	0.37
	LDL cholesterol	0.002 (0.009)	0.25	0.81
	HDL cholesterol	0.0043 (0.04)	0.11	0.91
Blueberry group—Men	Waist circumference	0.007 (0.04)	0.18	0.86
	Glucose	0.002 (0.06)	0.03	0.98
	Systolic blood pressure	−0.022 (0.01)	−0.19	0.86
	Diastolic blood pressure	−0.0003 (0.01)	−0.02	0.99
	Triglycerides	0.0025 (0.002)	1.21	0.28
	Total cholesterol	−0.004 (0.007)	−0.56	0.60
	LDL cholesterol	−0.003 (0.005)	−0.05	0.96
	HDL cholesterol	0.077 (0.04)	2.12	0.09
Women	Waist circumference	−0.029 (0.10)	−1.91	0.15
	Glucose	−0.198 (0.01)	−2.05	0.13
	Systolic blood pressure	0.0052 (0.003)	1.65	0.20
	Diastolic blood pressure	0.045 (0.008)	5.38	0.01
	Triglycerides	−0.015 (0.003)	−4.87	0.02
	Total cholesterol	0.011 (0.004)	2.98	0.06
	LDL cholesterol	−0.001 (0.003)	−0.42	0.68
	HDL cholesterol	−0.117 (0.02)	−5.83	0.01

* *p* values ≤ 0.06 are bolded; ** adjusted for age, total calorie intake and compliance.

## Abbreviations

The following abbreviations are used in this manuscript:

BMI	body mass index
CKD	chronic kidney disease
CVD	cardiovascular disease
SUA	serum uric acid
